# Determinants of timely initiation of breast milk and exclusive breastfeeding in Malawi: a population-based cross-sectional study

**DOI:** 10.1186/s13006-019-0232-y

**Published:** 2019-08-16

**Authors:** Owen Nkoka, Peter A. M. Ntenda, Victor Kanje, Edith B. Milanzi, Amit Arora

**Affiliations:** 1 Institute for Health Research and Communication (IHRC), Lilongwe, Malawi; 2 School of Public Health, College of Public Health, Taipei Medical University, Taipei, Taiwan; 3 School of Public Health and Family Medicine, Department of Public Health, College of Medicine, University of Malawi, Zomba, Malawi; 4 Institute for Risk Assessment Sciences (IRAS), Department of Environmental Epidemiology and Veterinary Public Health, Utrecht University, Utrecht, Netherlands; 5 School of Science and Health, Western Sydney University, Campbelltown, NSW Australia; 6 Discipline of Child and Adolescent Health, Sydney Medical School, Faculty of Medicine and Health, Westmead, NSW Australia; 7 Oral Health Services, Sydney Local Health District and Sydney Dental Hospital, NSW Health, Surry Hills, NSW Australia; 8 Translational Health Research Institute, Western Sydney University, Campbelltown, NSW Australia

**Keywords:** Early initiation of breastfeeding, Exclusive breastfeeding, Malawi

## Abstract

**Background:**

Breastfeeding practices such as early initiation of breast milk and exclusive breastfeeding are key to the reduction of childhood morbidity and mortality. Despite the importance of these practices, rates of timely initiation of breastfeeding and exclusive breastfeeding remain suboptimal in many sub-Saharan countries. This study aimed to examine the determinants of early initiation of breastfeeding and exclusive breastfeeding in the first 5 months in Malawi.

**Methods:**

This study used the 2015–16 Malawi Demographic and Health Survey data. A total of 6351 children born during the last 24 months and 1619 children aged 0–5 months at the time of the survey were analyzed for early initiation of breastfeeding and exclusive breastfeeding outcomes, respectively. Socio-demographic and socio-economic factors including individual, household and community-level factors were tested for association with early initiation of breastfeeding and exclusive breastfeeding using logistic regression models.

**Results:**

The proportion of timely initiation of breast milk and exclusive breastfeeding were 76.9 and 61.2%, respectively. Delivering at a health facility (adjusted odds ratio [aOR] 1.77, 95% confidence interval [CI] 1.10, 2.87), vaginal delivery (aOR 3.15, 95% CI 2.40, 4.13), and singleton births (aOR 1.96, 95% CI 1.20, 3.21) were independent factors associated with the increased likelihood of timely initiation of breastfeeding. Age of children was associated with increased odds of exclusive breastfeeding, with children aged 3–5 months being less likely to be exclusively breastfed (aOR 0.24, 95% CI 0.18, 0.31).

**Conclusions:**

Healthcare providers and programs aimed at increasing rates of early initiation of breastfeeding should take into consideration women at risk such as those giving birth through caesarean section, giving birth at home, and having multiple births. Further, women with children aged 3–5 months should be targeted with health promotion interventions for exclusive breastfeeding.

## Background

Appropriate breastfeeding practices are essential for the growth, survival, and development of infants [1]. Early initiation of breastfeeding (early initiation) is defined as feeding through mothers’ breast milk to newborn infants within the first hour of birth, while exclusive breastfeeding (EBF) refers to infants receiving breast milk only i.e. no other liquids (not even water) or solids, with the exception of oral rehydration salt solution, vitamins, mineral supplements or medicines [1, 2]. The benefits of breastfeeding within 1 h of birth and EBF to both the mother and child are well-documented such as the reduction of risk of gastrointestinal diseases and acute respiratory infections, and better intellectual and social development [3–7]. Globally, appropriate breastfeeding practices have the potential to prevent over 800,000 under-fives deaths annually [8]. It is also estimated that 22% of neonatal deaths could be prevented if infants are put to the breast within the first hour of birth [3]. In developing countries, an estimated 13% of all child deaths could be prevented if optimal breastfeeding levels were achieved [8]. Therefore, adherence to the aforementioned breastfeeding recommendations is critical for good health outcomes and child survival [2, 5].

Despite the evident benefits, the rates of early initiation and EBF globally remain low with approximately 50% of children under 2 years of age being breastfed within 1 h of birth [4, 6, 9]. Only 37% of infants under 6 months are exclusively breastfed in low and middle income countries [6]. In Africa, low prevalence of both early initiation and EBF have been reported [4, 5]. For instance, only 38.4% of mothers in Nigeria were reported to have initiated breastfeeding within the first hour of birth [10] while a 17% EBF rate was also reported [11].

In Malawi, breastfeeding is universal with approximately 98% of all children reported to have been breastfed at some point in time [12]. The Malawi National Nutrition Policy and Strategic Plan recommends key outputs in strengthening infant and young feeding practices in Malawi [13]. Example activities outlined in the policy include: the provision of training by service providers to orient expectant women on the benefits of both breastfeeding within 1 h of birth and EBF; and sensitization and awareness campaigns [13]. However, a decline in the EBF rate in the first 6 months was observed from 72% in 2010 to 61% in 2016 [12] thus highlighting the need to understand the factors associated with breastfeeding practices in Malawi.

Previous studies have reported inconsistent findings regarding the factors associated with early initiation of breast milk and exclusive breastfeeding practices [3, 4, 14–20]. For instance, although delivering at a health facility was found to be associated with increased likelihood for exclusive breastfeeding in Nepal [19], such an association was not reported in the Amibara district of northeastern Ethiopia [20]. These inconsistencies underscore the need for country-specific profiling of the determinants of early initiation and EBF. In Malawi, there is limited information regarding factors associated with early initiation and EBF practices.

Considering the declining EBF rates, the lack of research and inconsistent findings regarding factors associated with early initiation and EBF across countries, studies examining factors associated with optimal breastfeeding practices in Malawi are warranted. Therefore, using a nationally representative sample, this study sought to examine child-, maternal-, and health-related factors associated with early initiation and EBF in the first 5 months following birth in Malawi.

## Methods

### Study setting and data source

Malawi is located in southern-central Africa with an agriculture-dependent economy. The data were extracted from the 2015–16 Malawi Demographic Health Survey (MDHS) [12]. A total of 7970 children were included for analysis. The analysis for early initiation was restricted to the most recent birth in the past 2 years (*n* = 6351). However, analysis for EBF was restricted to the most recent birth and infants aged 0–5 months (*n* = 1619) [1].

### Sampling and data collection

The methodology, design and sampling methods of the MDHS have been detailed elsewhere [12]. Briefly, the survey used the 2008 Malawi Housing and Population census as its sampling frame. A two-stage cluster sampling design was employed with 850 clusters selected randomly and household listing conducted in the first stage. Using equal probability systematic selection, households from the chosen clusters were selected in the second stage.

Questionnaires, which were translated into two prominent local languages (Chichewa and Tumbuka), were administered to women of reproductive age (15–49 years) from the selected households via face-to-face interviews. The MDHS questionnaire collects data on a wide range of health indicators, including infant and young child feeding practices in addition to socio-demographic information.

### Measurements

#### Dependent variables (early initiation of breastfeeding, exclusive breastfeeding)

The dependent variable in this study was optimal breastfeeding measured using two binary variables namely “early initiation of breastfeeding” (early initiation) and “exclusive breastfeeding” (EBF). Early initiation was defined as children less than 24 months of age who were put to the breast within the first hour of birth [1, 12]. Those that reported having initiated breastfeeding within 1 h of birth were recorded as “1” while those that initiated breastfeeding after 1 h were recorded as “0”. Exclusive breastfeeding was defined as the number of infants aged 0–5 months (less than 6 months) who were fed exclusively with breast milk (including milk expressed or from a wet nurse) in the last 24 h [1, 12]. EBF allows the infant to receive oral rehydration salts, drops, syrups (vitamins, minerals, medicines) [1, 12]. Those who reported having fed their infant (0–5 months) with breast milk only or the allowed aforementioned liquids were recorded as “1” while those who had given infants other foods in addition to breast milk were recorded as “0”.

#### Independent variables

A number of child, maternal, and health-related independent variables were selected for analysis. Child factors included age in months (0–2, 3–5, and 6–23) and sex (male, female). Maternal factors included maternal age in years (15–24, 25–34, ≥35), marital status (married, unmarried), education (no formal education, primary education, secondary/post-secondary), and occupation (employed, unemployed). Wealth was calculated by the MDHS team using a principal component analysis model in which household items such as roofing materials and possession of bicycles were scored. The calculated scores were then divided into quintiles from poorest (lowest 20%) to richest (top 20%) [12]. In this study, the top 40% were categorized as rich, the middle 20% as middle class, and the bottom 40% were categorized as poor. Other maternal factors included region (northern, central, southern), place of residence (urban, rural), parity (primipara, secundipara, multipara), and media exposure measured by exposure to any of the following; newspaper, radio or television (yes, no). Health-related variables included place of delivery (health facility, non-health facility), number of antenatal visits, categorized as adequate (4 or more visits) or inadequate (fewer than 4 visits) [21, 22], antenatal care attendant, categorized as skilled (health professionals) or unskilled (traditional attendants/no one), birth attendant, also categorized as skilled (health professionals) or unskilled (traditional birth attendant/self/no one), mode of delivery (caesarean section, vaginal delivery), birth type (singleton, twin or multiple), and perceived size of the child at birth (small, average, large).

### Statistical analysis

All analyses were performed using Stata version 15.0 (Stata Corp LP, College Station, TX, USA) and considered the complex sample design. The “svy” command was used to adjust for cluster sampling design and sampling probabilities across clusters and strata. The distribution of participants according to early initiation and EBF status were analyzed using the Chi-squared tests. Unadjusted logistic models were used to examine the association between independent variables and early initiation and EBF, respectively. Variables with *p*-value ≤0.25 in the unadjusted model were manually included in the multivariate logistic models using purposeful selection method [23]. Adjusted odds ratio (aOR) and their 95% confidence intervals (CI) were used to report the strength of association between independent variables and early initiation and EBF, respectively. Sensitivity analysis was conducted to include only women who were tested for HIV for the purpose of controlling for HIV status and the results were fairly consistent. In this sensitivity analysis, HIV status remained insignificant and the sample was reduced to 2080 and 515 for EBF and early initiation, respectively. Therefore, we present results from the whole sample regardless of the availability of HIV status. The level of significance was set at *p* < 0.05 (two-tailed).

## Results

### Distribution of participants according to EIFB and EBF

Table 1 displays the characteristics of participants according to early initiation and EBF status. Out of 6351 last-born children born in the 2 years before the survey; 4889 (76.9%) were introduced to breastfeeding within the first hour of delivery. There was a significant (*p* < 0.05) difference between children who received breast milk within the first hour and those who did not in terms of infant age, maternal education, wealth, region, residence, parity, place of delivery, birth attendant, mode of delivery, birth type, and size of the child at birth.

**Table 1:** Distribution of study characteristics

Variable	Early initiation of breastfeeding (*n* = 6351)			Exclusive breastfeeding (*n* = 1619)		
	No (*n* = 1462) n (%)	Yes(*n* = 4889) n (%)	*p-*value^a^	No(*n* = 628) n (%)	Yes (*n* = 991) n (%)	*p*-value^a^
Child age (months)^b^			**0.021**			**< 0.001**
0 ~ 2	228 (28.0)	586 (72.0)		184 (22.6)	630 (77.4)	
3 ~ 5	179 (22.3)	626 (77.7)		444 (55.2)	361 (48.8)	
6 ~ 23	1055 (22.3)	3677 (77.7)		-	–	
Child sex			0.183			0.131
Male	704 (22.0)	2493 (78.0)		328 (41.1)	470 (58.9)	
Female	758 (24.0)	2396 (76.0)		300 (36.5)	521 (63.5)	
Woman’s age			0.518			0.056
15–24	675 (23.3)	2216 (76.7)		336 (41.4)	475 (58.6)	
25–34	582 (23.4)	1905 (76.6)		222 (37.8)	365 (62.2)	
≥35	205 (21.1)	768 (78.9)		70 (31.7)	151 (68.3)	
Marital status			0.443			0.559
Unmarried	218 (21.9)	782 (78.1)		88 (40.9)	127 (59.1)	
Married	1244 (23.2)	4107 (76.8)		540 (38.5)	864 (61.5)	
Education			**0.004**			**0.006**
No Education	160 (21.1)	600 (78.9)		80 (38.3)	127 (61.7)	
Primary Edu	927 (21.8)	3320 (78.2)		445 (41.8)	621 (58.2)	
Secondary +	375 (27.9)	969 (72.1)		103 (29.9)	243 (70.1)	
Occupation			0.346			0.869
Unemployed	448 (22.0)	1590 (78.0)		231 (38.5)	369 (61.5)	
Employed	1014 (23.5)	3299 (76.5)		397 (39.0)	622 (61.0)	
Wealth			**< 0.001**			0.059
Poor	639 (20.9)	2426 (79.1)		326 (42.4)	442 (57.6)	
Middle	253 (20.5)	978 (79.5)		116 (37.6)	193 (62.4)	
Rich	570 (27.7)	1485 (72.3)		186 (34.3)	356 (65.7)	
Region			**< 0.001**			0.053
Northern	142 (19.4)	593 (80.6)		83 (46.1)	97 (53.9)	
Central	740 (27.6)	1940 (72.4)		221 (34.8)	415 (65.2)	
Southern	580 (19.8)	2356 (80.2)		324 (40.3)	479 (59.7)	
Residence			**< 0.001**			0.060
Urban	317 (36.4)	554 (63.6)		71 (30.6)	161 (69.4)	
Rural	1145 (20.9)	4335 (79.1)		557 (40.2)	830 (59.8)	
Number of children			**0.018**			**0.019**
Primipara	449 (26.4)	1254 (73.6)		190 (43.1)	250 (56.9)	
Secundipara	281 (21.3)	1037 (78.7)		154 (42.7)	207 (57.3)	
Multipara	732 (22.0)	2598 (78.0)		284 (34.7)	534 (65.3)	
Media exposure			0.316			**0.037**
No	947 (22.5)	3259 (77.5)		433 (41.0)	623 (59.0)	
Yes	515 (24.0)	1629 (76.0)		195 (34.7)	368 (65.3)	
Place of delivery			**0.004**			0.873
Non-health facility	135 (30.6)	307 (69.5)		44 (37.9)	72 (62.1)	
Health facility	1327 (22.5)	4582 (77.5)		584 (38.8)	919 (61.2)	
Number of ANC visits			0.909			0.706
Inadequate	753 (22.9)	2531 (77.1)		352 (39.3)	543 (60.7)	
Adequate	709 (23.1)	2358 (76.9)		276 (38.2)	448 (61.8)	
ANC attendant			0.444			0.812
Unskilled	63 (21.0)	239 (79.0)		32 (40.3)	47 (59.7)	
Skilled	1399 (23.1)	4650 (76.9)		596 (38.7)	944 (61.3)	
Birth attendant			**0.035**			0.815
Unskilled	156 (28.0)	403 (72.0)		58 (37.8)	95 (62.2)	
Skilled	1306 (22.6)	4489 (77.4)		570 (38.9)	896 (61.1)	
Mode of delivery			**< 0.001**			0.085
Caesarean section	206 (48.7)	218 (51.3)		36 (29.6)	86 (70.4)	
Vaginal delivery	1256 (21.2)	4671 (78.8)		592 (39.5)	905 (60.5)	
Birth type			**< 0.001**			0.351
Twin/multiple	40 (40.7)	59 (59.3)		15 (48.8)	16 (51.2)	
Singleton	1422 (22.7)	4830 (77.3)		613 (38.6)	975 (61.4)	
Size of child at birth			**0.005**			0.463
Small	232 (23.6)	752 (76.4)		95 (35.4)	173 (64.6)	
Average	676 (20.9)	2553 (79.1)		320 (38.3)	514 (61.7)	
Large	554 (25.9)	1584 (74.1)		213 (41.2)	304 (58.8)	

Bold means *p* < 0.05

*secondary+* secondary or higher education, *ANC* Antenatal care

^a^Pearson’s chi-square test, ^b^age of exclusive breastfeeding sample = 0~5 months

Approximately 61.2% (991) children were exclusively breastfed. EBF was associated with a child aged 0–2 months, having a mother with secondary or higher education, and multiparous women (all *p* < 0.05, Table 1).

### Factors associated with early initiation of breastfeeding

Table 2 presents the adjusted and unadjusted odds ratios (aOR) for early initiation. Children aged 6–23 months had 32% (aOR 1.32, 95% CI 1.05, 1.66) higher odds of receiving breast milk in the first hour of birth compared to those aged 0–2 months. Compared to urban dwellers, rural dwellers had increased odds of initiating timely breastfeeding (aOR 1.98, 95% CI 1.50, 2.60). Secundipara mothers had increased odds (aOR 1.34, 95% CI 1.06,1.67) of initiating timely breastfeeding compared to primiparous women. Women who delivered at a health facility (aOR 1.78, 95% CI 1.10, 2.88), underwent vaginal delivery (aOR 3.15, 95% CI 2.40, 4.13), and had singleton births (aOR 1.96, 95% CI 1.19, 3.24)] were more likely to have had breastfeeding initiated in the first hour of birth compared to women who did not deliver at a health facility, those who delivered by caesarean section and those who had multiple births, respectively. Women from the central region had 41% (aOR 0.59, 95% CI 0.47, 0.76) reduced odds of early initiation of breastfeeding compared to those from the northern region.

**Table 2:** Factors associated with early initiation and exclusive breastfeeding in Malawi: results from unadjusted and adjusted logistic regression models

Variable	Early initiation of breastfeeding (*n* = 6351)				Exclusive breastfeeding (*n* = 1619)			
	OR (95% CI)	*p*-value	aOR^b^ (95% CI)	*p-*value	OR (95% CI)	*p*-value	aOR^c^ (95% CI)	*p-*value
Child age (months)^a^								
0 ~ 2	1.00		1.00		1.00		1.00	
3 ~ 5	1.35 (1.02, 1.80)	**0.038**	1.28 (0.95, 1.73)	0.104	0.24 (0.18, 0.30)	**< 0.001**	0.24 (0.18, 0.30)	**< 0.001**
6 ~ 23	1.35 (1.08, 1.70)	**0.008**	1.32 (1.05, 1.66)	**0.018**		–	–	–
Child sex								
Male	1.00		1.00		1.00		1.00	
Female	0.89 (0.76, 1.05)	0.183	0.88 (0.74, 1.04)	0.144	1.21 (0.94, 1.55)	0.131	1.14 (0.86, 1.50)	0.367
Woman’s age								
15–24	1.00				1.00		1.00	
25–34	0.99 (0.84, 1.18)	0.962			1.16 (0.90, 1.50)	0.237	0.83 (0.55, 1.26)	0.382
≥35	1.14 (0.88, 1.47)	0.327			1.52 (1.08, 2.16)	**0.017**	0.99 (0.54, 1.79)	0.967
Marital status								
Unmarried	1.00				1.00			
Married	0.92 (0.76, 1.13)	0.443			1.10 (0.79, 1.54)	0.560		
Education								
No formal Edu.	1.00		1.00		1.00		1.00	
Primary Edu	0.96 (0.73, 1.26)	0.757	1.02 (0.76, 1.35)	0.909	0.87 (0.61, 1.24)	0.431	0.91 (0.60, 1.38)	0.647
Secondary +	0.69 (0.51, 0.94)	**0.018**	1.05 (0.75, 1.48)	0.769	1.46 (0.94, 2.26)	0.090	1.59 (0.92, 2.76)	0.099
Occupation								
Unemployed	1.00				1.00			
Employed	0.92 (0.76, 1.10)	0.346			0.98 (0.76, 1.25)	0.869		
Wealth								
Poor	1.00		1.00		1.00		1.00	
Middle	1.02 (0.82, 1.27)	0.862	1.02 (0.81, 1.28)	0.896	1.22 (0.88, 1.69)	0.226	1.16 (0.81, 1.66)	0.405
Rich	0.69 (0.57, 0.83)	**< 0.001**	0.91 (0.74, 1.12)	0.363	1.41 (1.04, 1.91)	**0.026**	1.02 (0.71, 1.47)	0.911
Region								
Northern	1.00		1.00		1.00		1.00	
Central	0.63 (0.50, 0.79)	**< 0.001**	0.59 (0.47, 0.76)	**< 0.001**	1.60 (1.09, 2.34)	**0.015**	1.74 (1.19, 2.53)	**0.004**
Southern	0.98 (0.79, 1.21)	0.818	0.94 (0.75, 1.17)	0.578	1.27 (0.89, 1.78)	0.176	1.37 (0.97, 1.94)	0.077
Residence								
Urban	1.00		1.00		1.00		1.00	
Rural	2.17 (1.71, 2.75)	**< 0.001**	1.98 (1.50, 2.60)	**< 0.001**	0.66 (0.42, 1.02)	0.061	0.69 (0.42, 1.12)	0.130
Number of children								
Primipara	1.00		1.00		1.00		1.00	
Secundipara	1.32 (1.05, 1.65)	**0.015**	1.34 (1.06, 1.67)	**0.011**	1.02 (0.73, 1.43)	0.910	0.94 (0.66, 1.38)	0.799
Multipara	1.27 (1.05, 1.56)	**0.014**	1.21 (0.99, 1.51)	0.093	1.43 (1.08, 1.67)	**0.021**	1.54 (1.14, 3.00)	**0.009**
Media exposure								
No	1.00				1.00		1.00	
Yes	0.92 (0.78, 1.08)	0.316			1.31 (1.02, 1.69)	**0.037**	1.14 (0.85, 1.54)	0.379
Place of delivery								
Non-health facility	1.00		1.00		1.00			
Health facility	1.51 (1.14, 2.01)	0.004**	1.78 (1.10, 2.88)	**0.018**	0.96 (0.60, 1.54)	0.873		
Number of ANC visits								
Inadequate	1.00				1.00			
Adequate	0.99 (0.83, 1.17)	0.909			1.05 (0.82, 1.33)	0.706		
ANC attendant								
Unskilled	1.00				1.00			
Skilled	0.88 (0.64, 1.22)	0.444			1.07 (0.61, 1.87)	0.812		
Birth attendant								
Unskilled	1.00		1.00		1.00			
Skilled	1.33 (1.02, 1.74)	**0.035**	1.05 (0.66, 1.67)	0.825	0.95 (0.64, 1.42)	0.815		
Mode of delivery								
Caesarean section	1.00		1.00		1.00		100	
Vaginal delivery	3.52 (2.71, 4.59)	**< 0.001**	3.15 (2.40, 4.13)	**< 0.001**	0.64 (0.39, 1.07)	0.088	0.87 (0.50, 1.51)	0.624
Birth type								
Twin/multiple	1.00		1.00		1.00			
Singleton	2.33 (1.43, 3.79)	**0.001**	1.96 (1.19, 3.24)	**0.007**	1.52 (0.63, 3.67)	0.355		
Size of child at birth								
Small	1.00		1.00		1.00			
Average	1.17 (0.93, 1.46)	0.175	1.19 (0.95, 1.49)	0.126	0.89 (0.62, 1.29)	0.532		
Large	0.88 (0.69, 1.12)	0.319	0.93 (0.73, 1.20)	0.588	0.79 (0.53, 1.19)	0.258		

Bold means *p* < 0.05

*secondary+* secondary or higher education, *ANC* Antenatal care, *CI* Confidence interval, *OR* Odds ratio, aOR Adjusted odds ratio, ^a^age of exclusive breastfeeding sample = 0~5 months

^b^adjusted for child age, child sex, education, wealth, region, residence, number of children, place of delivery, birth attendant, mode of delivery, birth type, and size of child at birth

^c^adjusted for child age, child sex, woman’s age, education, wealth, region, residence, number of children, media exposure, and mode of delivery

### Factors associated with EBF

Child age, region, and parity were significantly associated with EBF. Children aged 3–5 months (aOR 0.24, 95% CI 0.18, 0.31) were less likely to be exclusively breastfed compared to those aged 0–2 months. Mothers from the central region had 74% (aOR 1.74, 95% CI 1.19, 2.53) increased odds of exclusively breastfeeding their children compared to those from the northern region. Finally, mothers who were multiparous were more likely (aOR 1.54, 95% CI 1.14, 3.00) to exclusively breastfeed their children compared to primiparous women (Table 2).

## Discussion

This is the first population-based study to examine determinants of early initiation of breast milk and exclusive breastfeeding in Malawi. The results reveal that, among others, health facility delivery, vaginal delivery, and singleton births had increased odds of children being breastfed within the first hour of birth. Further, children aged 3–5 months were less likely to be exclusively breastfed compared to those younger than 3 months. Regional variations were observed for both early initiation and EBF, with children from the central region exhibiting increased odds of EBF and reduced odds of early initiation compared to their counterparts from the northern region.

The current study demonstrated that in Malawi the rates of early initiation and EBF are 76.9 and 61.2%, respectively. Similar rates of EBF were observed in Gambia (60.2%) [24]. The World Health Organization considers early initiation rates ranging between 50 and 89% as good [25] and Malawi’s early initiation rate falls within this category. However, more needs to be done to graduate from good to very good (prevalence between 90 and 100%) [25]. Despite a relatively higher EBF rate compared to other sub-Saharan African countries [11], the rate has fallen from 71% in 2010. Thus, it is critical to discuss the factors associated with EBF in Malawi.

### Factors associated with early initiation of breastfeeding

Our study finding that rural residency was associated with increased odds of early initiation of breastfeeding is in contrast to some other studies [15, 20]. One study that similarly reported rural residents had increased odds of early initiation of breastfeeding was conducted in Saudi Arabia [26]. One possible explanation for this finding is that women in urban areas may be more often exposed to infant formula advertisements than their rural counterparts, which could increase the chances of bottle feeding rather than breastfeeding in the first hour of birth. However, more detailed research is warranted to understand this pattern [27, 28].

Secundipara women were associated with increased odds of breastfeeding initiation within 1 h of birth. Similar findings were reported in the Amibara district of northeastern Ethiopia where primipara women were less likely to initiate breastfeeding early [20]. Primiparous women may be associated with having little knowledge about pregnancy [29], while secundipara women may have the experience and knowledge gained from past pregnancies to know when exactly to initiate breastfeeding. Children from the central region were less likely to be breastfed early (within 1 h of birth). In Malawi, a higher percentage of educated women dwell in the northern region as compared to central region [12]. This might explain the current study’s observation that women from the central region were less likely to initiate breastfeeding within 1 h of birth as most of the women from this region may be less likely to have formal education compared to those from the northern region. Higher maternal educational levels have been associated with increased odds of having early initiation of breastfeeding elsewhere [19]. Therefore, government needs to deliver health promotion initiatives and educate women in the central region of Malawi.

In terms of the health-related factors, delivering at health facility, vaginal delivery, and being born singleton were associated with increased odds of early initiation of breastfeeding. This is consistent with other African studies [15, 20]. The benefits of delivering at heath facility include close monitoring by skilled attendants who may help the mother to initiate breastfeeding. In Malawi, the infant and young child feeding guidelines recommend that midwives or other skilled staff encourage early breastfeeding initiation [30]. In addition, the longer time taken to recover after caesarean section is thought to have an influence on early initiation of breastfeeding [31]. Further, delayed skin-skin contact due to prolonged mother-infant separation has been reported to be a reason for failure to initiate breastfeeding among women who give birth by caesarean section [32]. This might explain why in this study, consistent with previous research [18, 32], children born by vaginal delivery were more likely to be breastfed within 1 h of birth than those born by caesarean section. In addition, the birth of twins or other multiple births has been reported to be physically and mentally demanding compared to a singleton birth with subsequent effects observed on the timing of initiation of breastfeeding [33].

### Factors associated with EBF

Consistent with a study conducted in India [34], our results reveal that children aged 3–5 months were less likely to be exclusively breastfed compared to those aged 0–2 months. Mothers of older infants may feel that their children are ready to be introduced to complementary food unlike those children younger than 3 months. In Nigeria, a study reported an existent belief that after 3 months, breast milk is insufficient and therefore, other feeds are thought to be necessary in addition to the breast milk [35]. Therefore, educational messages aimed at improving exclusive breastfeeding should target mothers with children in this age group. Children from the central region in this study were more likely to be exclusively breastfed as compared to those from the northern region. As previously noted, a high proportion of women from the northern region in Malawi are educated [12] and therefore, are more likely to get better opportunities for employment. In Malawi, maternity leave for working mothers is for a period of 3 months which is inadequate to exclusively breastfeed children under the age of 6 months [36, 37]. Multiparity was associated with increased odds of exclusive breastfeeding. Higher parity may translate to experience in child rearing and thus, awareness of what is required in terms of EBF [35]. In contrast, a study in Bangladesh reported no significant association between parity and EBF [38].

A strength of this study is the use of a nationally representative sample, which enables the results to be generalizable to Malawian women. However, the study is subject to some limitations. The information collected about early initiation and EBF was based on women’s recall, and thus recall bias may have occurred. Limiting the sample to last born children in the last 2 years may have helped to minimize the bias. As early initiation and EBF practices were self-reported, social desirability bias may have occurred hence the results should be interpreted with caution. The cross-sectional design of the study could not allow causal inferences to be made.

## Conclusion

This study examined individual factors and health-related factors associated with early initiation of breastfeeding and exclusive breastfeeding in Malawi. Factors such as delivering at a health facility, vaginal delivery, singleton births, region, and number of children were found to be significantly associated with these breastfeeding practices in Malawi. Healthcare providers should pay attention to the risk groups namely: women delivering at home or by caesarean section and multiparous women, to increase the rate of early initiation of breastfeeding in Malawi. In addition, programs aiming at educating women about EBF are recommended. Future studies should consider examining community influences on optimal breastfeeding practices.

## Data Availability

The study used, with permission, data from the International Classification of Functioning, Disability, and Health (ICF). The data are publicly available upon request from the ICF on (https://dhsprogram.com/data/available-datasets.cfm).

## Abbreviations

aOR: adjusted odds ratio
CI: Confidence interval
EBF: Exclusive breastfeeding
MDHS: Malawi Demographic Health Survey
OR: Odds ratio
